# Genetic diversity analysis of *Dermacentor nuttalli* within Inner Mongolia, China

**DOI:** 10.1186/s13071-021-04625-5

**Published:** 2021-03-01

**Authors:** Zheng Gui, Lin Wu, Hao Cai, Lan Mu, Jing-Feng Yu, Shao-Yin Fu, Xiao-Yan Si

**Affiliations:** 1 Graduate School, Inner Mongolia Medical University, Hohhot, 010059 Inner Mongolia China; 2 Department of Parasitology, Inner Mongolia Medical University, Hohhot, 010110 Inner Mongolia China; 3 Inner Mongolia Academy of Agricultural & Animal Husbandry Science, Hohhot, 010031 Inner Mongolia China; 4 Inner Mongolia Center for Disease Control and Prevention, Hohhot, 010000 Inner Mongolia China

**Keywords:** Inner Mongolia, *Dermacentor nuttalli*, Haplotype, Genetic diversity

## Abstract

**Background:**

Ticks (Arthropoda, Ixodida), after mosquitoes, are the second most prevalent vector of infectious diseases. They are responsible for spreading a multitude of pathogens and threatening the health and welfare of animals and human beings. However, given the history of tick-borne pathogen infections in the Inner Mongolia Autonomous Region of China, surprisingly, neither the genetic diversity nor the spatial distribution of haplotypes within ticks has been studied.

**Methods:**

We characterized the haplotype distribution of *Dermacentor nuttalli* in four main pastoral areas of the Inner Mongolia Autonomous Region, by sampling 109 individuals (recovered from sheep) in April–August 2019. The 16S rRNA gene, cytochrome c oxidase subunit I (COI), and the internal transcribed spacer 2 region (ITS2) were amplified and sequenced from extracted DNA.

**Results:**

Twenty-six haplotypes were identified using 16S rRNA sequences, 57 haplotypes were identified with COI sequences, and 75 haplotypes were identified with ITS2 sequences. Among the three genes, total haplotype diversity was greater than 0.7, while total nucleotide diversity was greater than 0.06. Neutrality tests revealed a significantly negative Tajima’s D result, while Fu's Fs was not significantly positive. Fixation index values (F_ST_) indicated that the degree of genetic differentiation among some sampled populations was small, while for others it was moderate. Analysis of molecular variance (AMOVA) revealed that the variation within populations was greater than that among populations. The mismatch analysis of *D. nuttalli* exhibited double peaks.

**Conclusion:**

The genetic diversity of *D. nuttalli* populations in our region can likely adapt to different geographical environments, thereby leading to genetic diversity, and creating genetic differentiation among different populations. However, genetic differentiation is cryptic and does not form a pedigree geographical structure.
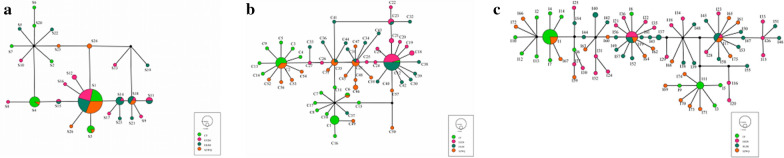

## Background

*Dermacentor nuttalli* (Acari: Ixodidae) is widely distributed across northern China, Russia and Mongolia [1], and is generally found in arid grasslands suitable for grazing cattle and sheep [2]. *D. nuttalli* is responsible for spreading a variety of diseases, including spotted fever, tick-borne *Rickettsia*, Crimean-Congo hemorrhagic fever and babesiosis [2–5], in addition to being an important vector of spirochetes and forest encephalitis [6, 7]. Humans may experience symptoms similar to viral infections after being bitten by ticks, including headaches, muscle pain, fevers, enlarged local lymph nodes or plaques, as well as severe liver and kidney damage and damage to the central nervous system [8, 9]. *D. nuttalli* is an important storage host and transmission medium of *Rickettsia*, resulting in the rapid spread of *Rickettsia* through bites and causing a variety of zoonotic diseases. As a result, *D. nuttalli* is quickly becoming one of the most dangerous tick species to public health in Inner Mongolia.

Yet, despite the increasing danger to the health and economy of the Inner Mongolia Autonomous Region, neither the genetic diversity nor the spatial distribution of haplotypes in *D. nuttalli* populations has been studied. In recent years, research into the genetic diversity within species and even between populations has become increasingly common [10–14]. Genetic diversity is affected by many biotic and abiotic factors, including natural selection, genetic variation and anthropogenic influences [15]. Ticks and pathogens have also been identified as influencing host evolution [16, 17]. Nevertheless, there remains a paucity of data for tick populations within China. Therefore, the goal of this study was to characterize the genetic diversity of various *D. nuttalli* populations to further our understanding not only of their diffusion, but also of their evolution, in order to protect the health of humans and animals. We hope these data will create a foundation for further study into the spread of tick-borne diseases in China and provide evidence for the origin and continued evolution of tick species.

We chose three gene regions for our study, as they are all easily amplified and commonly used in molecular systematics and population genetics [19–21]. While the 16S rRNA gene has a relatively slow rate of evolution [18], and the cytochrome c oxidase subunit I gene (COI) is a highly conserved region, they are both widely used in the study of intra- and interspecific genetic diversity, as well as in phylogenetic geography among different geographical populations [22, 23]. In comparison, the internal transcribed spacer 2 region (ITS2) evolves rapidly, resulting in rich polymorphism. As a result, it is commonly used to study historical population dynamics of species with close interspecific relationships [24–28]. In this study, we analyzed the haplotype distribution of the 16S rRNA, COI and ITS2 genes within individuals of *D. nuttalli* sampled from four main pastoral areas of the Inner Mongolia Autonomous Region.

## Methods

### Sample collection

**Fig. 1:**
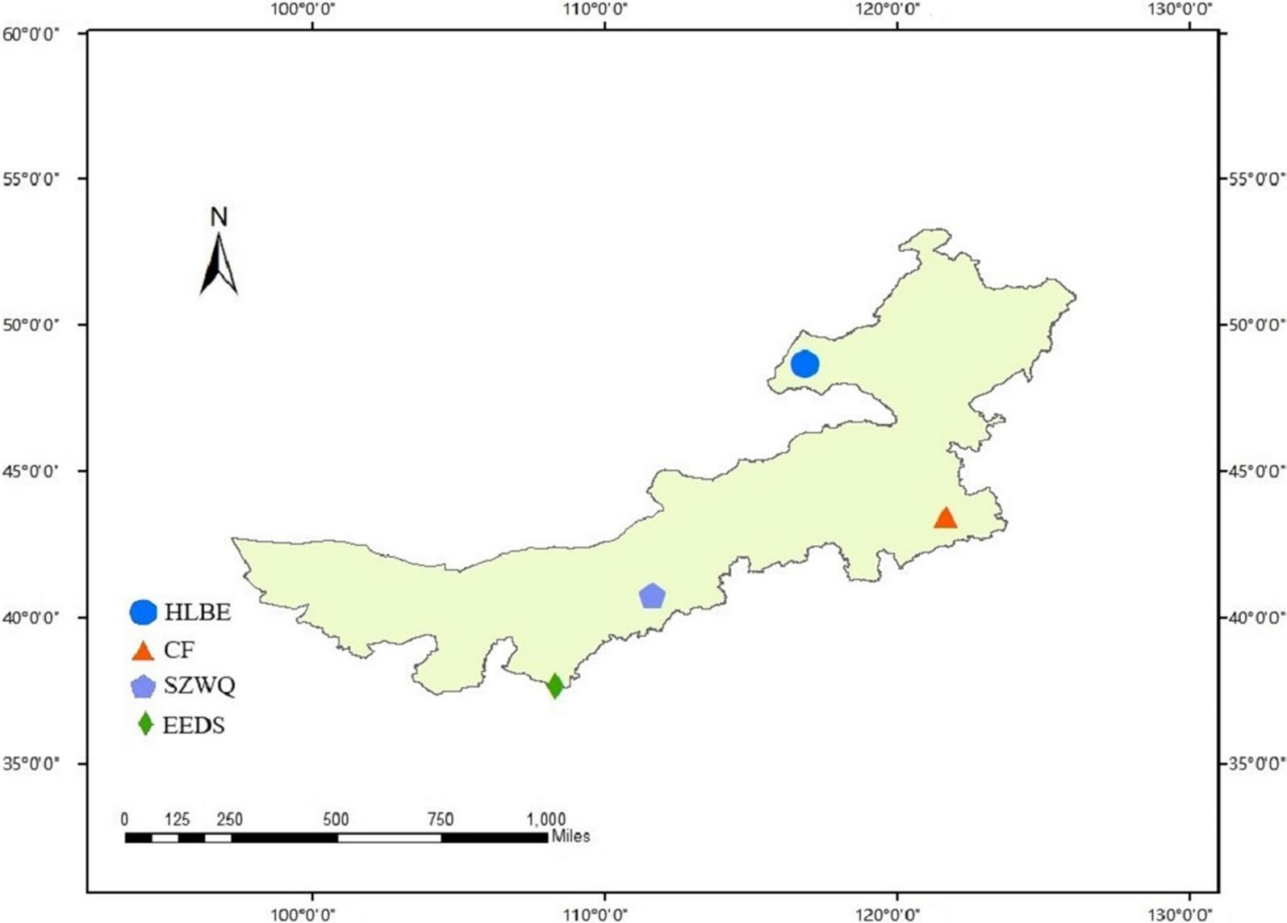
Collection site map. Samples of *Dermacentor nuttalli* in four regions of Inner Mongolia were collected. Each color corresponds to different collection regions in Inner Mongolia, and each graphic represents the approximate geographical coordinates of the location in the map for each collection site

A total of 109 *D. nuttalli* individuals were sampled from Chengchuan Town, early Banner of Etoke Banner, Ordos City (EEDS), Siziwang Banner, Hohhot City (SZWQ), the Bayan WenduSumu area, Arukorqin Banner, Chifeng City (CF) and Xinbarhu right Banner, Hulun Buir City (HLBE), Inner Mongolia. All ticks were recovered from sheep using tweezers and rubber gloves between April and August 2019 (Table 1, Fig. 1) and were stored at −80 °C.

**Table 1:** Sample information of *Dermacentor nuttalli* populations

Location	Collection time	Area code	Longitude	Latitude	Samples
ChiFeng	2019.4	CF	121°64′	43°46′	29
Siziwang Banner	2019.5	SZWQ	111°63′	40°81′	25
Hulun Buir	2019.8	HLBE	116°82′	48°67′	29
Ordos	2019.7	EEDS	108°32′	37°70′	26

### DNA extraction

Each *D. nuttalli* individual was washed in three sterile water baths followed by one absolute ethanol bath, air dried and collected in a sterile EP tube. Each tick was then placed inside a 1.5 ml sterile micro-tube containing sterile micro-beads (Jingxin, Shanghai, China) with a 2:1 ratio of two small steel micro-beads (2 mm diameter) to one large steel micro-bead (4 mm diameter). Samples were cooled in liquid nitrogen for 2 minutes and crushed by a tissue grinder (Jing Xin, Shanghai, China) for two cycles (2 minutes, frequency of 60). The tubes were then briefly centrifuged at a speed of 12,000 rpm and then extracted using the TIANamp Genomic DNA Kit (TIANGEN, Beijing, China) following the protocol for tissue extraction. The final elution volume was 70 μL, and the samples were stored at −20 °C [29].

### Amplification and sequencing

To analyze the genetic structure of our the four populations, we amplified the 16S rRNA, COI and ITS2 gene regions. The primer templates for all three genes were downloaded from the NCBI database. The upstream and downstream primers of 16S rRNA gene were located in the v4 and v7 variable regions, respectively. The COI and ITS2 amplification products were approximately full length (Oligo7). The primer sets of the 16S rRNA, COI and ITS2 gene sequences were synthesized by Shanghai Sangon Biotechnology (Shanghai, China). The 40 μL PCR reaction mixture contained 20 μL Taq PCR Master Mix (Sangon), 1 μL of each primer, 16 μL ddH_2_O and 2 μL of DNA from each sample. Details about PCR primer pairs, size of the amplification (base pairs [bp]), and annealing temperatures are presented in Table 2. Negative control samples (sterile double-distilled water) were included in each PCR reaction. After electrophoresis, gel imaging was used to determine whether amplification was successful, and if the amplified band was the target band, it was retained. If the target band was bright and free, it was sent directly for sequencing, while other bands were excised, purified using a TIANGEN Gel DNA recovery Kit (TIANGEN, Beijing, China) and cloned using the pGM-T Easy Vector System.

**Table 2:** Primers of PCR and amplification conditions

Gene target	Primer sequence (5'**~**3')	Amplification size (bp)	Annealing temperature (°C)
D16SF	ATGAAAATCTTTAAATTGCTG	472	50
D16SR	CCTCATTCTCATCGGTCT		
DCO1F	CGAATAGAACTTAGCCAACCT	1210	50
DCO1R	AATAACGACGGGGTATTCCT		
DITS2F	TCCGTCGACTCGTTTTGACC	1040	57
DITS2R	GGATACATCGCTTTCGCCCAT		

### Data analysis

Nucleotide sequences from the three amplified regions (16S rRNA, COI and ITS2) were spliced by SeqMan 7.1. Our sequences were compared to those previously uploaded to GenBank using the Basic Local Alignment Sequence Tool (BLAST) search engine. In order to ascertain the degree of genetic diversity among these four *D. nuttalli* populations, we estimated a number of parameters under a range of neutrality tests (including Tajima’s D, Fu and Li’s and Fu’s tests) in DnaSP 5.10, including the number of segregating sites, the average number of nucleotide differences, the number of haplotypes, haplotype diversity and the distribution pattern of DNA variation [30, 31]. The genetic differences among populations were calculated by the fixed index (F_ST_). In order to determine the relationship between haplotypes [32], PopART [Population Analysis with Reticulate Trees] version 1.7 was used to construct TCS haplotype network maps. The phylogenetic tree was constructed for all haplotypes of the 16S rRNA, COI and ITS2 gene sequences of the four geographic populations of *D. nuttalli* in MEGA7.0 using the adjacency method. We downloaded the 16S rRNA, COI and ITS2 gene sequences of the three genes from different geographic populations in China (NCBI), and the higher homology of *D. nuttalli* with *Haemaphysalis longicornis* was chosen as the outgroup. The model was the Kimura two-parameter model, and the stability of the tree was evaluated by 1000 bootstrap repeats [33–35].

## Results

### Population genetic analysis

#### 16S rRNA

**Fig. 2.**
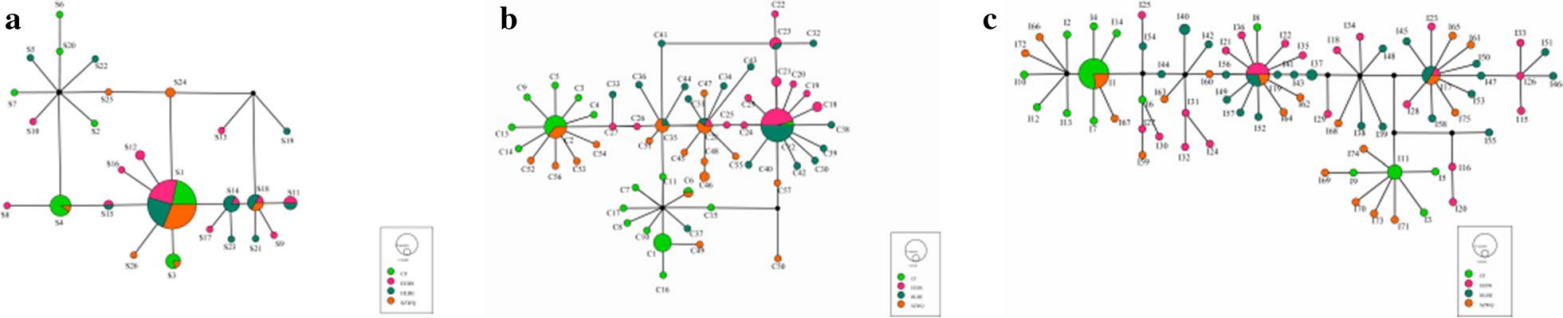
**a** TCS haplotype network of *Dermacentor nuttalli* based on 16S rRNA genes from four different populations in Inner Mongolia. **b** TCS haplotype network of *Dermacentor nuttalli* based on COI from four different populations in Inner Mongolia. **c** TCS haplotype network of *Dermacentor nuttalli* based on ITS2 from four different populations in Inner Mongolia

With the 16S rRNA sequences, the final alignment consisted of 664 base pairs, with 453 variable sites. We detected 26 haplotypes (GenBank accession numbers MW486582–MW486607) that contained seven shared haplotypes (S1, S3, S4, S11, S14, S15, S18), with shared haplotypes varying in certain genetic differentiation among populations. The most frequent haplotype detected was S1, with 55 sequences (50.5% of all sequences). S1 was located in the center of the haplotype network diagram and was a shared haplotype of the four geographic populations, indicating that these populations were relatively stable and could adapt to different environments. The shared haplotype was the dominant haplotype among groups and likely could adapt to the environment more successfully than the remaining 19 unique haplotypes (Fig. 2a). Total average haplotype diversity (Hd) was 0.732 and varied among populations (ranging from 0.5 to 1), and total nucleotide diversity was 0.06091. Diversity values were calculated for each sampled locality. HLBE was the locality with the highest haplotype diversity (*h* = 0.946), while EEDS had the lowest diversity (*h* = 0.849). Regarding nucleotide diversity, the lowest diversity was found in SZWQ (pi = 0.00721), and the highest (pi = 0.10363) in CF (Table 3).

**Table 3:** Summary statistics for polymorphisms and neutrality tests of the 16S rRNA gene from *Dermacentor nuttalli* from Inner Mongolia

	*n*	Ht	S	Hd	Pi	Tajima’s	Fu and Li’s D	Fu and Li’s F	Fu’s Fs
CF	29	13	205	0.909	0.10363	−1.07674	1.43476	0.70664	10.425
EEDS	26	14	236	0.849	0.06060	−2.63037***	−4.38637	−4.49895**	3.448
HLBE	29	19	175	0.946	0.07681	−1.98627*	−0.00382	−0.78554	0.557
SZWQ	25	15	15	0.913	0.00721	−0.99819	−0.31899	−0.61416	−8.569*
Total	109	26	181	0.732	0.06091	−2.00467*	−1.45181	−2.03427	3.884

*n*, number of samples; Ht, number of haplotypes; S, number of polymorphic sites; Hd, haplotype diversity; Pi, nucleotide diversity

* *p* < 0.05; **p < 0.01; ***p < 0.001

**Fig. 3:**
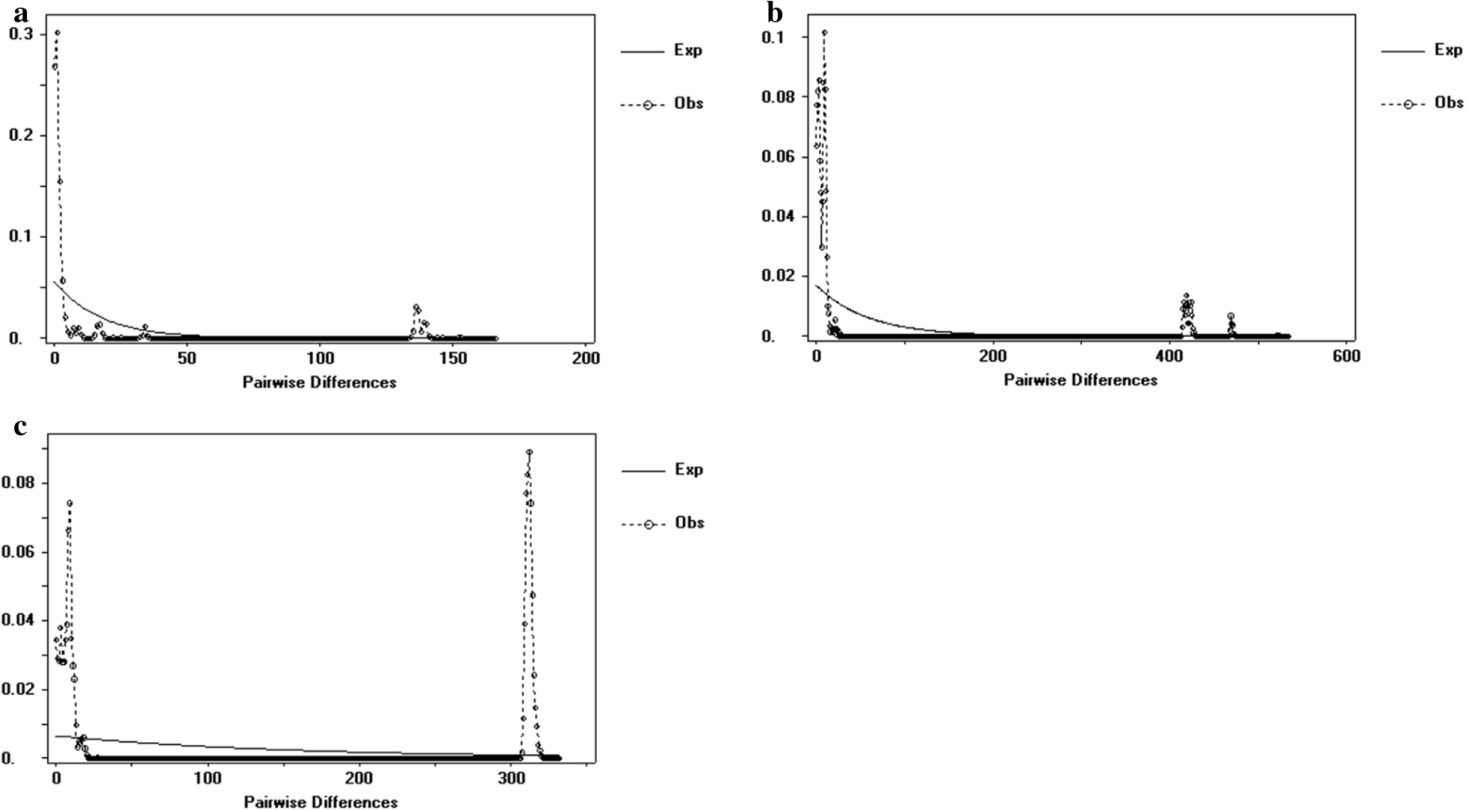
**a** Mismatch distribution analysis for the *Dermacentor nuttalli* groups based on 16S rRNA. **b** Mismatch distribution analysis for the *Dermacentor nuttalli* groups based on COI. **c** Mismatch distribution analysis for the *Dermacentor nuttalli* groups based on ITS2

Negative neutrality results obtained for most of the locations analyzed indicated an excess of recently derived haplotypes. While Tajima's D values were significant, Fu’s Fs values were not significant, also indicating that the population had not experienced expansion recently. The F_ST_ value reflects the degree of genetic differentiation between two populations, representing the allelic variation between the populations, which was inversely proportional to gene flow. The F_ST_ value between CF and SZWQ was greater than 0.05, indicating that there was moderate genetic differentiation among populations, with a small degree of gene flow. The F_ST_ values among the other regions were less than 0.05, indicating that the genetic differentiation among these populations was very small, likely as a result of high gene flow (Table 4). The results of analysis of molecular variance (AMOVA) indicated that the population variation of *D. nuttalli* mainly arises from within the population, and the genetic differentiation between populations is very small (Table 5). The mismatch analysis chart exhibited double peaks, indicating that the four geographic populations did not experience rapid population expansion (Fig. 3a).

**Table 4:** F_ST_ values among different groups of *Dermacentor nuttalli* based on the 16S rRNA gene

Locality	CF	EEDS	HLBE	SZWQ
CF				
EEDS	0.00169			
HLBE	−0.00708	−0.02164		
SZWQ	0.06478	0.0599	0.03312

**Table 5:** AMOVA of the 16S rRNA gene of the *Dermacentor nuttalli* population

Source of variation	d.		Sum of squares	Variance components	Percentage of variation	Fixation Index FST
Among population	3		31.322	0.07694Va	0.91	
Within population	105		876.457	8.34721Vb	99.09	0.00913
Total	108		907.78	8.42415

#### Cytochrome c oxidase subunit I (COI)

In the COI sequences, the final alignment consisted of 1267 base pairs, with 992 variable sites. A total of 57 haplotypes (GenBank Accession Numbers MW507375-MW507431) contained 6 shared haplotypes (C2, C6, C12, C23, C28, C35). The most frequent haplotype detected was C12, with a total of 24 sequences (22% of all sequences), although C35 was located in the center of the network. Given that C35 possessed the greatest number of connections and was most closely related to other haplotypes, it is likely an ancient haplotype (Fig. 2b). Total average haplotype diversity (Hd) was 0.936, while total nucleotide diversity was 0.07402. EEDS was the locality with the highest haplotype diversity (*h* = 0.997), while SZWQ had the lowest diversity (*h* = 0.957). Regarding nucleotide diversity, the lowest diversity was found in EEDS (pi = 0.00698) and the highest (pi = 0.16498) in CF (Table 6).

**Table 6:** Summary statistics for polymorphisms and neutrality tests of COI from *Dermacentor nuttalli* from Inner Mongolia

	*n*	Ht	S	Hd	Pi	Tajima’s	Fu and Li’s D	Fu and Li’s F	Fu’s Fs
CF	29	23	538	0.968	0.16498	0.30721	1.39599*	1.22059	5.122
EEDS	26	25	39	0.997	0.00698	−1.67142	−1.21492	−1.59944	−20.394
HLBE	29	24	546	0.963	0.04256	−2.78277***	−5.18100**	−5.18100**	−0.910
SZWQ	25	17	568	0.957	0.05704	−2.68241***	−4.77294**	−4.82784**	3.932
TOTAL	109	57	646	0.936	0.07402	−2.16243**	−3.90850**	−3.74087**	1.765

*n*, number of samples; Ht, number of haplotypes; S, number of polymorphic sites; Hd, haplotype diversity; Pi, nucleotide diversity. *p < 0.05; **p < 0.01; ***p < 0.001

As was the case with 16S rRNA, neutrality results revealed non-significant Tajima's D values and significant Fu's Fs values, confirming that the population had not experienced recent expansion. The F_ST_ value between CF and EEDS was the highest (F_ST_ = 0.15940), while the F_ST_ values for CF, SZWQ and HLBE were all greater than 0.05, indicating that there was moderate genetic differentiation among populations with a small degree of gene flow. The F_ST_ values among the three regions of SZWQ, EEDS and HLBE were all less than 0.05, indicating that the genetic differentiation among these populations was very small, likely as a result of high gene flow (Table 7). The results of AMOVA analysis and mismatch analysis were consistent with the 16S rRNA results (Table 8, Fig. 3b).

**Table 7:** F_ST_ values among different groups of *Dermacentor nuttalli* based on COI

Locality	CF	EEDS	HLBE	SZWQ
CF				
EEDS	0.1594			
HLBE	0.09702	−0.0044		
SZWQ	0.0836	0.0433	0.01367

**Table 8:** AMOVA of COI of *Dermacentor nuttalli* population

Source of variation	Degrees of freedom	Sum of squares	Variance components	Percentage of variation	Fixation Index FST
Among population	3	302.83	2.72298Va	9.21	
Within population	105	2819.069	26.84828Vb	90.79	0.09208
Total	108	3121.899	29.57126		

#### Internal transcribed spacer 2 (ITS2)

The final alignment of ITS2 sequences consisted of 1689 base pairs, with 1172 variable sites. Seventy-five haplotypes (GenBank accession numbers MW477806–MW477880) were obtained through ITS2 gene analysis, including three shared haplotypes. The most frequent haplotype detected was I1, represented by 17 sequences (14.8% of all sequences). I19 was in the center of the network map, while other shared haplotypes formed small cluster centers. The various clusters were surrounded, which may be related to the close geographic distance. There were no shared haplotypes in CF or HLBE, indicating that there was genetic differentiation between these two geographical populations (Fig. 2c). Total average haplotype diversity (Hd) was 0.966, while the total nucleotide diversity was 0.2619. EEDS was the locality with the highest haplotype diversity (*h* = 0.997), while CF had the lowest diversity (*h* = 0.931). Regarding nucleotide diversity, the lowest diversity was found in CF (pi = 0.21148) and the highest (pi = 0.28319) in SZWQ (Table 9).

**Table 9:** Summary statistics for polymorphisms and neutrality tests of ITS2 from *Dermacentor nuttalli* from Inner Mongolia

	*n*	Ht	S	Hd	Pi	Tajima’s	Fu and Li’s D	Fu and Li’s F	Fu’s Fs
CF	29	18	432	0.931	0.21148	1.40295	1.26459	1.55123*	14.588
EEDS	26	25	507	0.997	0.25278	−2.26714*	0.76842	1.48191	0.398
HLBE	29	25	435	0.988	0.26002	3.32432***	1.36722	2.39290**	3.638
SZWQ	25	22	417	0.987	0.28319	−2.91418**	1.22568	−2.08443**	3.104
TOTAL	109	75	368	0.966	0.26119	−2.92651**	−0.21650	1.42638	3.338

*n*, number of samples; Ht, number of haplotypes; S, number of polymorphic sites; Hd, haplotype diversity; Pi, nucleotide diversity. *p < 0.05; **p < 0.01; ***p < 0.001

Neutrality results were the same as that of 16S rRNA and COI. The F_ST_ values for CF and SZWQ, and CF and HLBE were all greater than 0.05, and the F_ST_ values of other regions were all less than 0.05, indicating that the genetic differentiation among these populations was very small, likely as a result of high gene flow (Table 10). The results of AMOVA analysis and mismatch analysis were consistent with 16S rRNA and COI results (Table 11, Fig. 3c).

**Table 10:** F_ST_ values among different groups of *Dermacentor nuttalli* based on ITS2

Locality	CF	EEDS	HLBE	SZWQ
CF				
EEDS	0.03084			
HLBE	0.10340	−0.01727		
SZWQ	0.05265	−0.02979	−0.02935

**Table 11:** AMOVA of ITS2 of *Dermacentor nuttalli* population

Source of variation	Degrees of freedom	Sum of squares	Variance components	Percentage of variation	Fixation Index FST
Among population	3	356.803	1.60792Va	2.09	
Within population	105	7894.041	75.18134Vb		
Total	108	8250.844	76.78926	97.91	0.02094

### Phylogenetic analysis

#### 16S rRNA

**Fig. 4:**
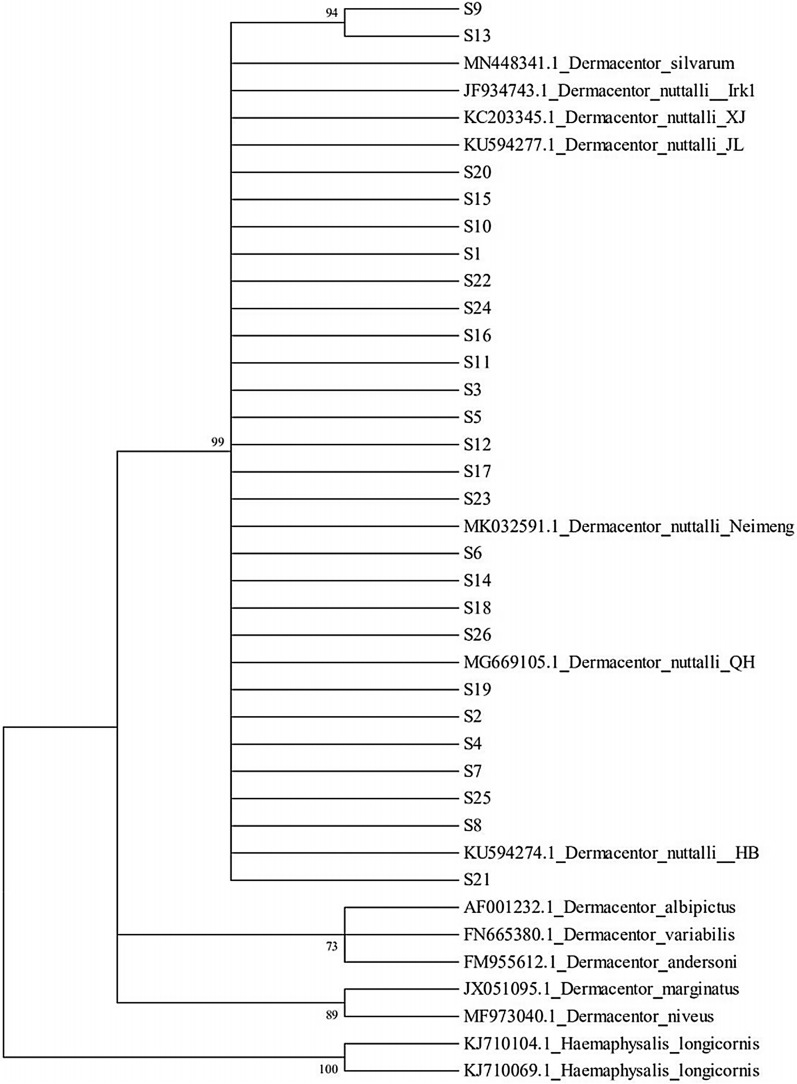
Phylogenetic tree based on the 16S rRNA gene of *Dermacentor nuttalli*

We found that the inner populations of *D. nuttalli* were clustered into one group, and the four geographic populations were mixed, with no obvious geographical differentiation structure. This implies that the genetic evolution among populations was not closely related to the geographical structure. Genetic analysis found that *D. nuttalli* and *D. silvarum* were closely related, and others were distributed in the periphery (Fig. 4).

#### Cytochrome c oxidase subunit I (COI)

**Fig. 5:**
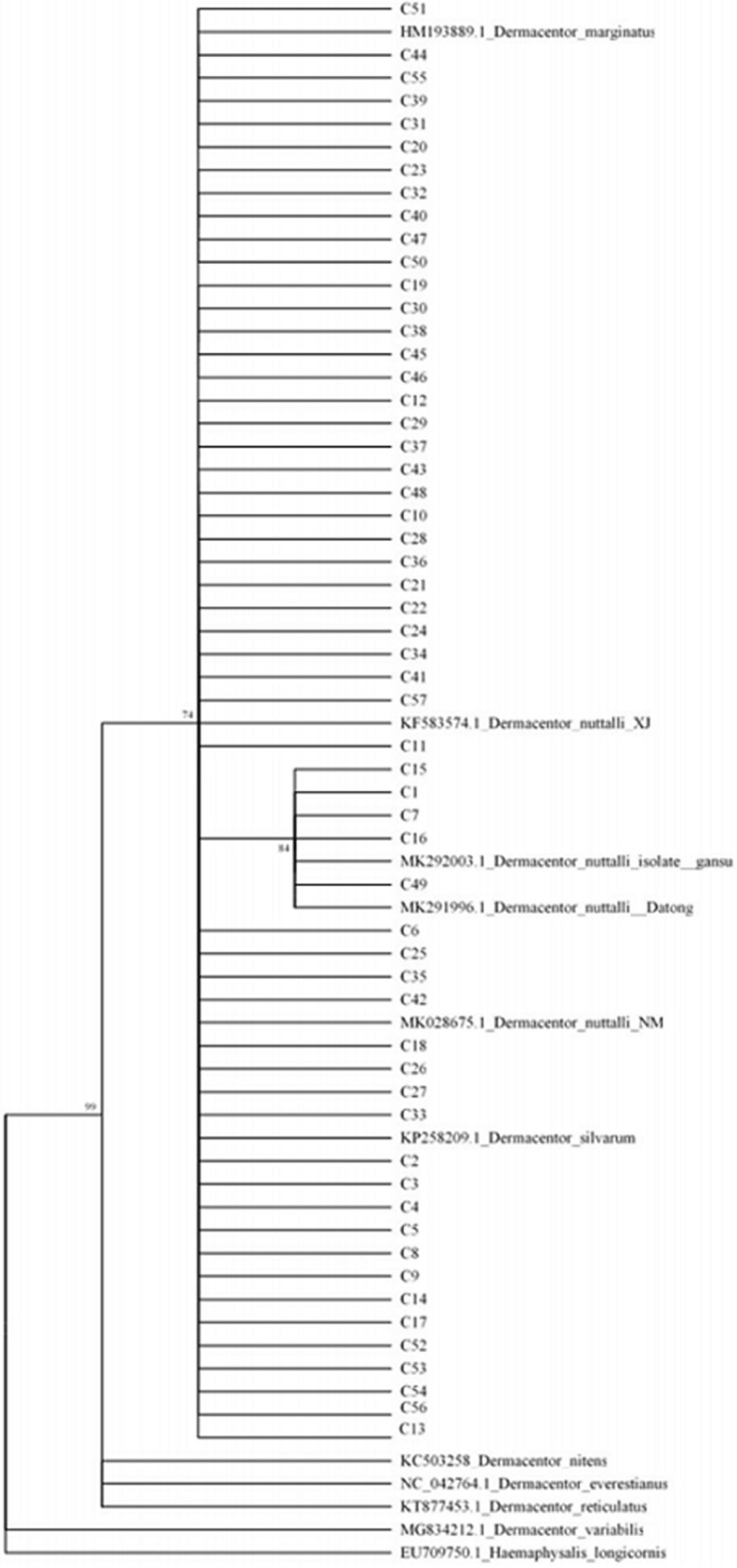
Phylogenetic tree based on COI of *Dermacentor nuttalli*

As in the case of 16S rRNA analysis, the haplotypes were gathered as a branch and mixed with each other. However, an additional phylogenetic tree revealed that *D. nuttalli* was related to *D. silvarum* and *D. marginatus* (Fig. 5).

#### Internal transcribed spacer 2 (ITS2)

**Fig. 6:**
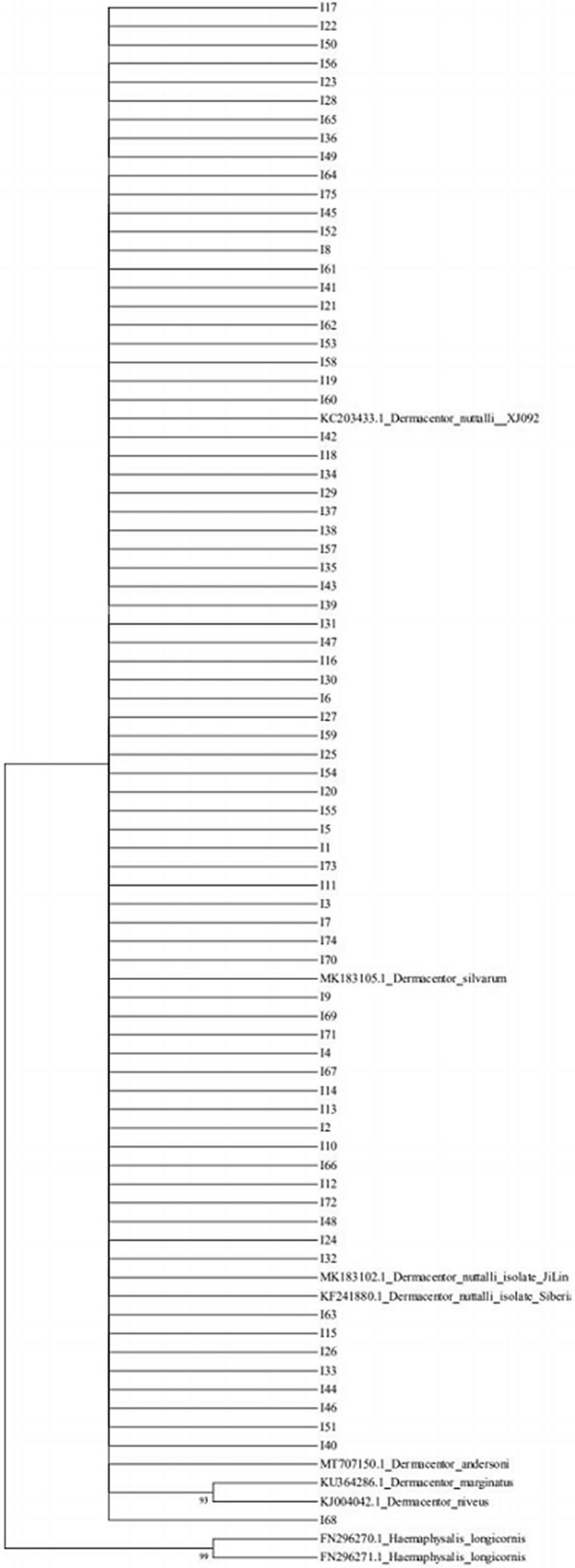
Phylogenetic tree based on ITS2 of *Dermacentor nuttalli*

The phylogenetic tree based on ITS2 gene sequences was identical to that created using the 16S rRNA sequences (Fig. 6).

## Discussion

This is the first report on the genetic structure of *Dermacentor nuttalli*, which is the dominant tick species in the Inner Mongolia Autonomous Region [36]. Haplotype diversity (Hd) and nucleotide diversity (Pi) are two important indicators to measure the diversity of a species population [37]. In this study, the genetic diversity of *D. nuttalli* populations was characterized among four different geographical populations in the Inner Mongolia Autonomous Region with three genes, revealing high haplotype diversity and high nucleotide diversity.

Our results indicate that only a few shared haplotypes were present across our four sampled populations, but that these shared haplotypes were also highly abundant. Shared haplotypes can persist for long periods in a population and can adapt to different environments [38]. While most of the haplotypes detected in our study were exclusive, indicating genetic differentiation among populations, the rich haplotype diversity and high genetic diversity indicate that *D. nuttalli* may have the ability to adapt to different environments within its large geographical range in Inner Mongolia. Our haplotype network diagram revealed highly connected shared haplotypes, indicating that not only was there frequent gene communication, but that there was also no effect of geographical isolation.

The neutral test results of Tajima's D and Fu's Fs can be used to analyze the historical dynamics of the population. If both are significantly negative, it means that the population has experienced rapid population expansion in history. Otherwise, there is no population expansion, and the population may differentiate. Therefore, all three genes exhibited genetic differentiation with no population expansion. The mismatch analysis maps are both bimodal, with no peak, which is consistent with the results of the neutrality test. F_ST_ values represent the genetic differentiation index of populations, which can measure the genetic differentiation among different populations [39]. In our study, the varying degrees of genetic differentiation among our four populations are likely determined by the different evolution rates of the three genes, but overall, the degree of genetic differentiation was small, with high gene flow present among populations. The phylogenetic tree and haplotype network illustrated that the haplotypes were not distributed according to the corresponding geographical units, and that the haplotypes of each geographical population were mixed. Therefore, *D. nuttalli* populations within Inner Mongolia did not conform to geographical isolation, and the reason for the formation of this structure was the combination of multiple factors.

In a study in Eastern Siberia, the genetic specificity and phylogeny of *D. nuttalli* across four sites characterized with 16S rRNA and ITS2 obtained only 11 haplotypes, which is significantly fewer than in our study [2]. While the documented population variation in Eastern Siberia was smaller, with lower diversity of haplotypes, the phylogenetic analysis was consistent with ours, in that the closest relative of *D. nuttalli* was *D. silvarum* and the next closest related species was *D. marginatus*. In this study, only four areas with *D. nuttalli* from Inner Mongolia were selected, which did not cover the distribution area of *D. nuttalli* in China. Further studies should increase the geographic sampling range to lay a foundation for controlling the further spread of tick-borne diseases and provide more information with which to study the origin and evolution of ticks.

## Conclusion

The 16S rRNA gene, COI and ITS2 were used to study the genetic diversity of *D. nuttalli* sampled from four geographic localities in Inner Mongolia. The genetic diversity of *D. nuttalli* populations in the Inner Mongolia Autonomous Region reflects adaptation to different geographical environments, and the adaptability of different populations resulted in genetic diversity that led to genetic differentiation among them. However, the genetic differentiation among populations was cryptic and did not form a pedigree geographical structure. The genetic diversity was related not only to gene mutation, but to natural selection and other environmental factors [40]. It was also likely related to the migratory ability, strong adaptability, diversity of hosts, environment and climate, habitat fragments and human activities. The data in this study documented the genetic diversity of ticks in Inner Mongolia and provided enhanced scientific understanding of the evolution of ticks.

## Data Availability

All datasets have been included with this article and our sequences have been deposited within GenBank (accession numbers MW486582–MW486607 for 16S rRNA, accession numbers MW507375–MW507431 for COI and accession numbers MW477806–MW477880 for ITS2).

## Abbreviations

COI: Cytochrome c oxidase subunit I
ITS2: Internal transcribed spacer 2
PCR: Polymerase chain reaction
*D. nuttalli*: *Dermacentor nuttalli*
*D. silvarum*: *Dermacentor silvarum*
*D. marginatus*: *Dermacentor marginatus*
AMOVA: Analysis of molecular variance
